# Multi-dimensional analysis of the global burden of colorectal cancer disease from 1990 to 2021 and prediction of future trends: A comprehensive study based on the GBD database

**DOI:** 10.1371/journal.pone.0337216

**Published:** 2025-12-10

**Authors:** Yilan Sun, Guangyi Zhu, Dongbo Lian, Buhe Amin, Guangzhong Xu, Jing Wang, Nengwei Zhang, Dezhong Wang

**Affiliations:** Surgery Centre of Diabetes Mellitus, Capital Medical University Affiliated Beijing Shijitan Hospital, Beijing, China; Sichuan University, CHINA

## Abstract

**Background:**

Colorectal cancer (CRC) is one of the malignancies with high morbidity and mortality rates worldwide, and its disease burden continues to increase with population aging and changes in lifestyle and dietary habits. Based on the Global Burden of Disease database (GBD), this study analyzed trends in global CRC incidence, deaths, and disability-adjusted life years (DALYs) from 1990 to 2021, and explored health inequalities across countries and regions.

**Methods:**

This study used data from the GBD Database 2021 to quantify the contribution of population aging, population growth, and epidemiological changes to the burden of CRC. Health inequalities were assessed on a global scale through the Slope index (SII) and concentration index (CI), and the potential room for improvement in the control of DALYs burden in CRC was assessed by countries using frontier analysis. The future disease burden of CRC was predicted based on a Bayesian Age-Period-cohort model (BAPC).

**Results:**

Worldwide, the incidence, death and DALYs burden of CRC increased significantly, mainly driven by population aging and population growth. Areas with high socio-demographic index (SDI) have significantly reduced the burden of disease through epidemiological changes, while the burden remains higher in areas with low SDI. Health inequalities have improved, but areas with low SDI still face a higher burden of disease. Frontier analysis shows that there is still much room for improvement in CRC prevention and control in countries with high SDI. Projections show that despite the decline in CRC deaths, the number of cases of CRC is expected to continue to increase due to the impact of population aging and population growth.

**Conclusions:**

Population aging and growth drive the global CRC burden increase. Low – SDI regions’ epidemiological changes have limited impact. Future policies should focus on low – SDI areas’ early prevention and screening and optimize resource allocation.

## 1 Introduction

Colorectal Cancer (CRC) is a major global public health challenge, and its disease burden ranks first among malignant tumors of the digestive system [1,2]. Epidemiological data show that CRC is prone to occur in the rectum and sigmoidorectal junction, and its age-standardized incidence continues to increase in most countries [3,4]. According to the International Agency for Research on Cancer’s 2022 Global Cancer Statistics Report, CRC has become the third most common malignancy and the second most cancer-related cause of death worldwide [5].

Over the past three decades, the epidemiological characteristics of CRC have evolved significantly. This is despite the fact that high-income countries have achieved some age-group reduction in incidence through screening programmes [6]. However, the overall global incidence continues to rise, driven by aging populations, westernized diets (high red meat intake, low dietary fiber), and sedentary lifestyles [7–9]. It is worth noting that the burden of disease is distributed with significant geographic heterogeneity: declining standardized mortality rates in countries with high sociodemographic indices (SDI) are in sharp contrast to rapid growth in countries with low and medium SDI [10], suggesting complex associations between socioeconomic factors (e.g., access to care, screening coverage) and disease outcomes [11].

The study showed that the disability-adjusted life years (DALYs) of CRC increased by 191.12% between 1990 and 2019, with attributions for metabolic risk (high BMI) and behavioral risk (smoking, alcohol) of 8.3% and 13.3%, respectively [12–14]. However, existing studies are insufficient to analyze the driving mechanism of regional differences, especially the differentiation effect of the contribution of aging, population growth and epidemiological changes in different SDI regions has not been quantified. Based on the Global Burden of Disease (GBD) database, this study quantifies the contribution of population growth, aging and epidemiological changes to the increase of cases through decomposition analysis. Combined with the theory of social determinants of health, the dynamic evolution of health inequality under SDI stratification was analyzed. Frontier analyses were further introduced to assess the effectiveness of disease control in countries and to predict future disease burden trends using Bayesian age-period-cohort (BAPC) models. The results of this study aim to reveal the “efficiency-equity” tradeoff mechanism of CRC prevention and control, and provide an evidence-based basis for optimizing tertiary prevention strategies and formulating differentiated cancer prevention and control policies.

## 2 Materials and methods

### 2.1 Data sources

The data of this study were derived from the GBD 2021 database. In terms of data coverage and stratification, the database includes 204 countries and regions worldwide, which are further categorized into 21 GBD regions based on geographical proximity, homogeneity in socioeconomic development, and consistency in disease epidemiological characteristics. Additionally, stratification was performed using the SDI, calculated as (Life Expectancy Index×Income Index×Education Index)^(1/3). In this study, SDI was divided into five tiers (low, low-middle, middle, high-middle, and high) using quintile classification to analyze the association between socioeconomic development level and the burden of CRC. Regarding core indicators: incidence rate refers to the ratio of new CRC cases to the exposed population in a specific group during the same period; mortality rate is the ratio of CRC deaths to the exposed population in the same period; and DALYs is a comprehensive indicator reflecting the impact of diseases on both life expectancy and quality of life, calculated as the sum of Years of Life Lost (YLLs) due to premature death and Years Lived with Disability (YLDs). To eliminate the impact of differences in population age structure, all rate-based indicators were age-standardized using the GBD 2021 standard population structure, resulting in the ASIR, ASMR, and ASDR. Data quality control followed a three-step process: “multi-source data integration - consistency verification - uncertainty assessment”. All data were obtained via the official GBD data query tool (https://vizhub.healthdata.org/gbd-results/).

**Table:** 

GBD Estimate	Cause of death or injury
Measure	Incidence, DALYs, Deaths
Metric	Number, Rate
Cause	Colon and rectum cancer
Location	Select all countries and territories
Age	Age-standardized
Sex	Both
Year	1999–2021

### 2.2 Research methods

This study adopted a multi-dimensional, progressive analytical framework, with all analyses conducted using R 4.3.0 software. The demographic decomposition model aimed to decompose the total changes in global CRC incidence, mortality, and DALYs from 1990 to 2021. The process involved: first, defining basic variables such as population size, crude rates, and age-standardized rates for the baseline year (1990) and target year (2021); second, calculating the total change; third, quantifying the population growth effect, population aging effect, and epidemiological change effect respectively; finally, calculating the contribution of each factor as the ratio of the single-factor effect value to the total change. Meanwhile, a “year-by-year recursive decomposition method” was used for sensitivity analysis to verify the stability of the results.

Health inequality assessment was conducted using a combination of the SII and CI. SII was based on robust weighted least squares regression, with regional SDI values as independent variables, CRC-ASDR as the dependent variable, and regional population size as weights. The SII value reflects the absolute change in ASDR for each 1-unit increase in SDI. Robustness was verified using the “single-region exclusion method”. CI was calculated based on the Lorenz curve, adjusted by population weighting, with values ranging from −1–1. The combination of SII and CI clarifies both the direction and magnitude of inequality, addressing the limitations of using a single indicator.

For prevention and control efficiency analysis, a SDI-ASDR performance frontier was constructed using Locally Weighted Scatterplot Smoothing (LOESS). A population-weighted scatter plot was created with SDI values of countries/regions as independent variables and ASDR as the dependent variable. A LOESS regression curve with a span of 0.75 was fitted, and the minimum ASDR boundary at the same SDI level was defined as the performance frontier. Prevention and control efficiency was quantified by the “effective difference” — the difference between the actual ASDR and the frontier ASDR. A positive difference indicates that efficiency is below the optimal level, while a value close to 0 indicates that efficiency reaches the optimal level for the corresponding SDI. The fitting effect of the frontier was verified using 5-fold cross-validation (RMSE < 10%).

Trend prediction analysis was based on the BAPC model, which assumes that CRC incidence/mortality risk is jointly driven by three effects: age effect, period effect, and cohort effect. A Poisson regression model was used as the basis, and the “second-order difference zero-mean constraint” was applied to address the identifiability issue of the model. Non-informative priors were used for precision parameters, and normal priors were used for intercept terms. Parameters were estimated using the Integrated Nested Laplace Approximation (INLA) algorithm. Finally, the posterior distributions and 95% UIs of CRC incidence and mortality rates for global and SDI-stratified regions were output, providing a forward-looking basis for prevention and control planning.

### 2.3 Statistical methods

All statistical analysis and data visualization were performed using R software (version 4.2.3) and JD_GBDR (version 2.32). Descriptive statistics were performed on morbidity, mortality, DALYs and age-standardized data. The results were expressed by means and 95% uncertainty interval. In trend analysis, P-values less than 0.05 were considered statistically significant.

The data used in this study are from the publicly available GBD database, and all data are aggregated statistics from de-identified populations, so there are no ethical approval requirements for human subjects.

## 3 Results

### 3.1 Breakdown of driving factors for changes in disease burden

**Fig 1:**
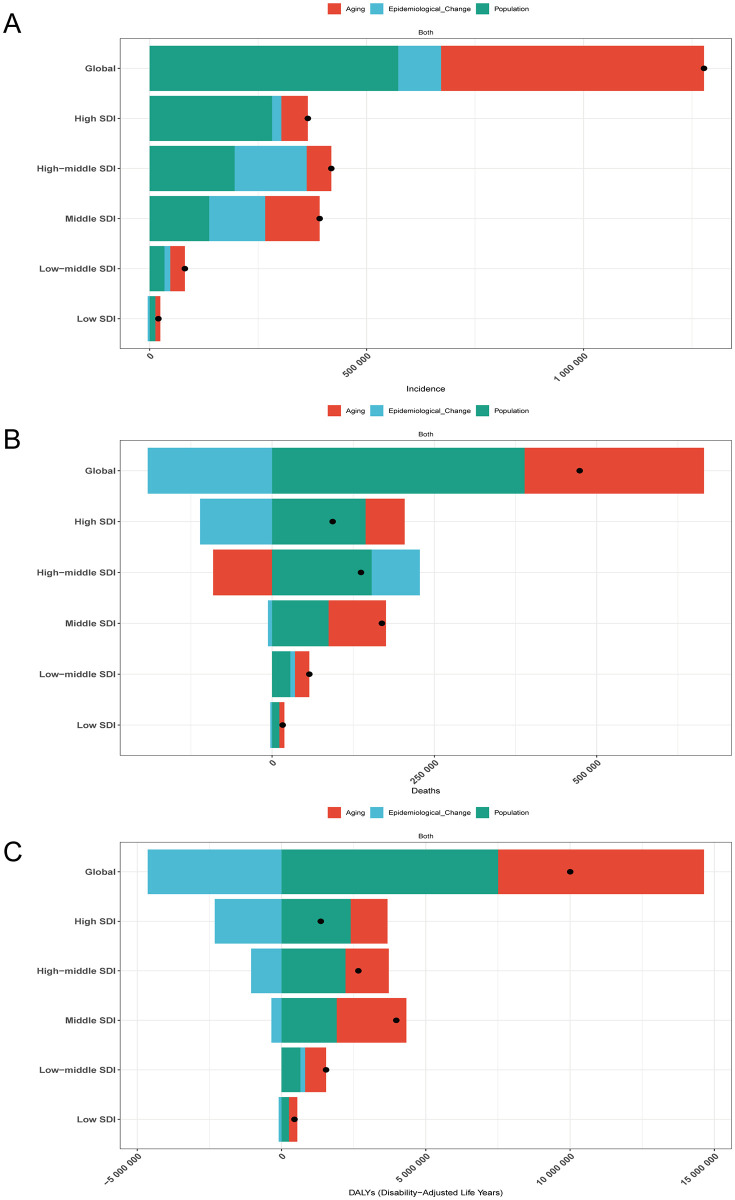
Contributions of aging, population growth, and epidemiological changes to CRC incidence (A), mortality (B), and DALYs (C) from 1990 to 2021 in global and SDI regions. The black dots represent the total effect values of the combined contributions of these three factors. SDI: sociodemographic indices; DALYs: disability-adjusted life years.

Population aging and population growth contributed 47.43% and 44.85%, respectively, to the global increase in the incidence of CRC from 1990 to 2021, while epidemiological changes contributed only 7.71%. The SDI stratification showed significant regional heterogeneity: 77.42% of the increase in incidence in high SDI areas was driven by population growth, while 57.23% of the increase in low SDI areas was attributed to aging (Fig 1A). The typical paradox is found in high-income North America, where the positive effect of population growth (+104.76%) is strongly counterbalanced by the negative inhibition of epidemiological change (−63.59%). In Western and Eastern Europe, the changing population structure has led to an unexpected reduction in the burden of disease due to aging. (−12.61% and −37.68%, respectively) (Table 1). The increase in global deaths was mainly due to population growth (82.07%) and aging (58.34%), while improvements in epidemiology resulted in a decrease in mortality, contributing −40.41%. Population growth continued to be an important cause of the increase in deaths, especially in high SDI and medium to high SDI areas, contributing 154.22% and 112.12%, respectively. Epidemiological changes show a different story, but in high and medium SDI regions, epidemiological improvements significantly offset potential growth (contribution of −118.74% and −3.73%, respectively). Regional analysis showed that the burden of death in Western Europe, high-income North America and Asia-Pacific was reduced by 305.35%, 339.10% and 21.34% through epidemiological optimization (S1 Table), but the expansion of population base was still the leading driving factor (contribution rate 364.02%, 341.61% and 59.85%) (Fig 1B). In the global DALYs growth, aging (71.29%) and population growth (75.05%) were synergistically driven, and the contribution rate of epidemiological improvement was −46.34%. SDI stratification showed that the contribution rate of epidemiological effect was as low as −169.84% in high SDI areas, while positive risk accumulation was shown in low and medium SDI areas (+10.39%), suggesting insufficient coverage of prevention and control measures. It is worth noting that although low SDI countries experience rapid population growth (contributing 57.28%), the driving intensity of aging on DALYs in low SDI countries (64.26%) is still significantly higher than that in middle and high SDI countries (56.34%), highlighting the double pressure of disease burden transfer (Fig 1C) (Table 1) (S2 Table).

**Table 1:** Decomposition analysis of inequality in CRC incidence counts from 1990−2021 by global and SDI regions.

Location	Overll difference	Aging	Population	Epidemiological Change	Aging Percentage	Population Percentage	Epidemiological Change Percentage
Global	1277559.72	605974.81	573040.15	98544.75	47.43	44.85	7.71
High SDI	364520.02	61066.93	282207.14	21245.96	16.75	77.42	5.83
High-middle SDI	418660.5	56907.67	196027.93	165724.89	13.59	46.82	39.58
Middle SDI	391758.16	125585.18	137416.55	128756.42	32.06	35.08	32.87
Low-middle SDI	81130.5	33620.04	34172.58	13337.88	41.44	42.12	16.44
Low SDI	20177.54	11548.43	13058.96	−4429.85	57.23	64.72	−21.95
Eastern Europe	41501.66	−15635.84	35699.03	21438.46	−37.68	86.02	51.66 Central Europe
Central Europe	43684.63	−171.16	24017.12	19838.67	−0.39	54.98	45.41
Oceania	284.64	177.33	125.99	−18.68	62.3	44.26	−6.56
East Asia	519843.74	190686.28	144441.89	184715.56	36.68	27.79	35.53
Central Asia	2712.8	1356.16	2946.53	−1589.89	49.99	108.62	−58.61
Southeast Asia	87620.77	34668.38	25004.03	27948.36	39.57	28.54	31.9
High-income North America	77036.28	45316.05	80704.54	−48984.31	58.82	104.76	−63.59
Central Latin America	36919.21	14575.82	8483.18	13860.2	39.48	22.98	37.54
Australasia	11437.5	5741.71	7628.37	−1932.59	50.2	66.7	−16.9
High-income Asia Pacific	127733.7	30826.03	57230.94	39676.73	24.13	44.8	31.06
Caribbean	12274.63	2425.8	5351.08	4497.75	19.76	43.59	36.64
Western Europe	131530.44	−16581.08	131729.63	16381.89	−12.61	100.15	12.45
Southern Latin America	13764.32	3570.11	7946.55	2247.66	25.94	57.73	16.33
Andean Latin America	6549.68	2986.68	2269.23	1293.77	45.6	34.65	19.75
North Africa and Middle East	48518.47	26859.65	18806.2	2852.61	55.36	38.76	5.88
South Asia	56976.93	30521.31	24232.62	2223	53.57	42.53	3.9
Tropical Latin America	34401.43	14771.16	11226.09	8404.18	42.94	32.63	24.43
Western Sub-Saharan Africa	7273.73	4527.93	4224.33	−1478.54	62.25	58.08	−20.33
Southern Sub-Saharan Africa	5107.1	2327.85	2527.36	251.89	45.58	49.49	4.93
Central Sub-Saharan Africa	2615.54	1941.19	1455.68	−781.33	74.22	55.65	−29.87
Eastern Sub-Saharan Africa	9772.55	4365.2	6778.09	−1370.74	44.67	69.36	−14.03

### 3.2 Evolution of health inequality trends

**Fig 2:**
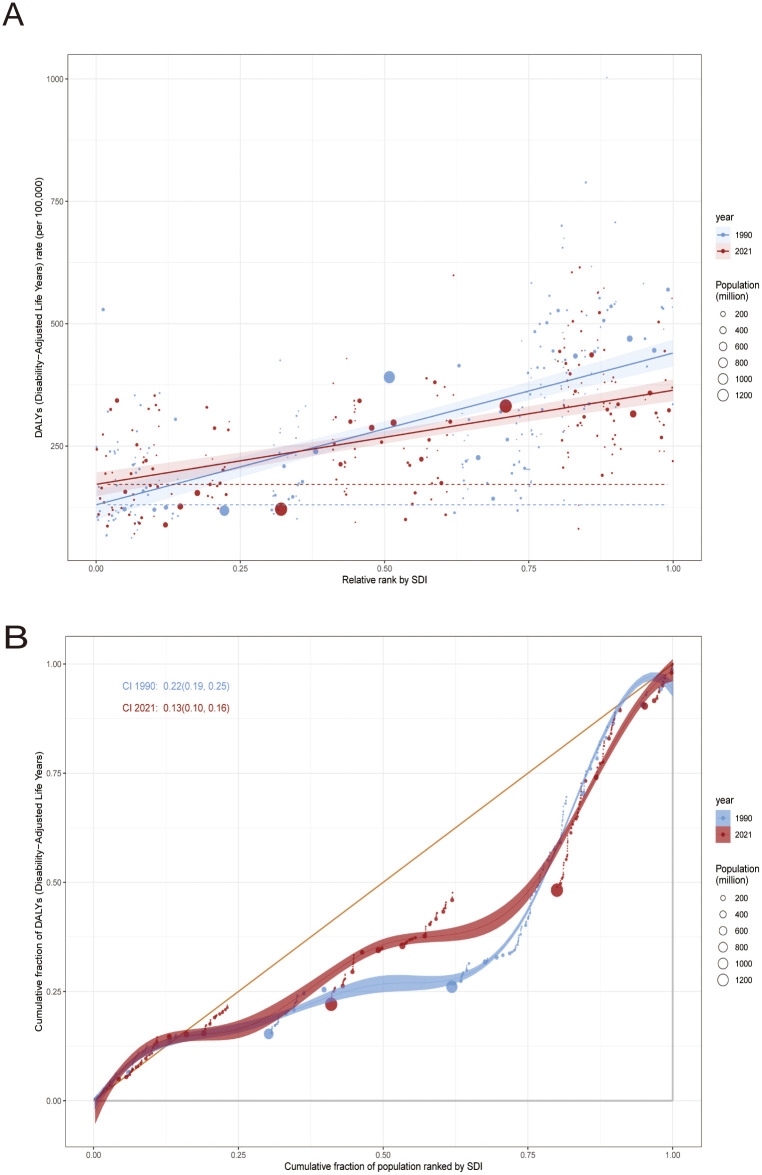
Comparison of inequality analysis and CI of global CRC disease burden in 1990 and 2021. CI: concentration index; CRC: Colorectal cancer.

As shown in Fig 2A, the absolute health inequality in ASDR for CRC shows a significant decreasing trend. In 1990, the SII value was 310 (95% CI: 262–358), indicating that the ASDR decreased by 310 units for every 1 unit increase in SDI, revealing that the disease burden borne by low SDI areas was about 3.1 times that of high SDI areas. By 2021, the SII has fallen to 192 (95% CI: 153–231), a 38.1% decrease, reflecting the reduction in absolute regional disparities by global health interventions. Nevertheless, the current SDI gradient is still significantly negatively correlated with the ASDR, and the ASDR in low SDI areas is consistently higher than that in high SDI areas.

Socio-economic relative health inequalities also show improvement characteristics (Fig 2B). A 1990 CI value of 0.22 (95% UI: 0.19–0.25) suggests that the DALYs burden of CRC is systematically concentrated in the low SDI population (CI > 0 suggests a “pro-affluenza” distribution). By 2021, the CI drops to 0.13 (95% UI: 0.10–0.16), a decrease of 40.9%, indicating an improvement in the fairness of resource allocation. Although both absolute and relative inequality indicators show an improvement trend between 1990 and 2021, DALYs rates remain higher in areas with low SDI than areas with high SDI. This persistent disparity may be due to multiple barriers such as differences in access to screening technologies, inadequate palliative care coverage, and lagging tumor registry systems, highlighting structural imbalances in the global CRC prevention and control system.

### 3.3 Frontier analysis of disease control efficiency

**Fig 3:**
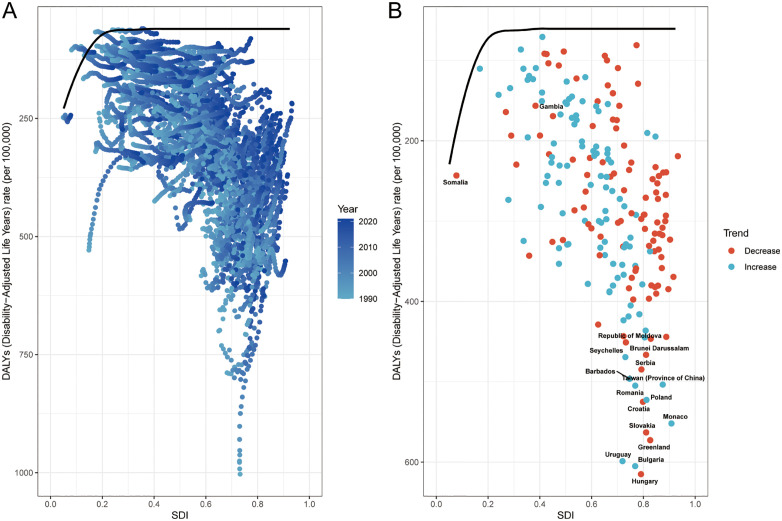
Frontier analysis of CRC DALYs rates based on SDI in 204 countries and regions worldwide from 1990 to 2021. SDI: sociodemographic indices; CRC: Colorectal cancer; DALYs: disability-adjusted life years.

From 1990 to 2021, the ASDR of CRC showed a decreasing trend in most countries or regions, especially in countries with high SDI, such as Norway and Switzerland. However, some low SDI countries are still showing an upward trend, such as the Republic of Togo. There is a nonlinear negative correlation between SDI and ASDR. The higher the SDI, the lower the ASDR generally (Fig 3A). By constructing the SDI-ASDR performance Frontier model, disease control efficiency was quantified in 204 countries and regions (Fig 3B). The 15 countries with the largest performance gaps (effective variance 390.32 to 554.19) include Moldova (390.32), Seychelles (408.45), Brunei Darussalam (405.70), Serbia (423.95), Barbados (435.74), Taiwan, China (442.81), Romania (4) (44.28), Poland (461.90), Croatia (464.28), Monaco (491.44), Slovakia (502.34), Greenland (512.14), Uruguay (538.26), Bulgaria (544.42), and Hungary (554.19), reflecting insufficient efficiency in the allocation of health resources. It is worth noting that high SDI countries such as Monaco (498.76) and Norway (472.15) are also included, revealing structural deficiencies such as insufficient coverage of early screening and uneven access to treatment despite their advanced medical systems.. In contrast, low SDI countries such as Somalia (effective difference 0.20) and Gambia (10.10) show minimal deviations (<5%) from the frontier value, indicating that they have achieved near theoretical optimal disease control within resource constraints through low-cost strategies such as universal primary prevention and synergistic prevention and control of infectious diseases and tumors. Such cases provide a feasible paradigm for “lean control” in resource-limited areas (Fig 3B).

### 3.4 Future development prediction of global morbidity and mortality based on BAPC effect

**Fig 4:**
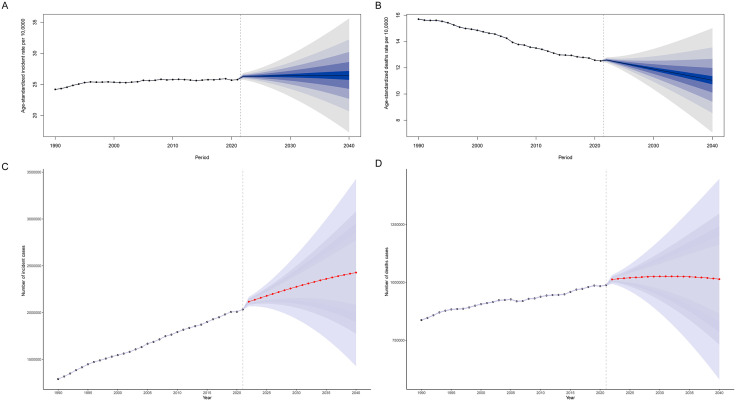
BAPC projection of global colorectal cancer incidence and death rates (number) through 2040. BAPC: Bayesian Age-Period-Cohort.

BAPC analysis predicts that by 2040, the age-standardized incidence of CRC will increase to 26.45 per 100,000 people (95%UI: 15.56–37.33) and the number of cases will increase to 2,427,475 (95%UI: 1,427,942−3,427,008) (Figs 4A, C). The ASMR will decrease to 11.05 per 100,000 population (95%UI: 6.33–15.76), but the number of deaths is projected to increase to 1,013,886(95%UI:581,378−1,446,393)(Figs 4B, D). For males, the incidence increased to 35.51 per 100,000, while the ASMR decreased to 19.41 per 100,000. In females, the incidence rate decreased to 19.02 per 100,000 and the mortality rate to 7.84 per 100,000 (S1 Fig). It is worth noting that the period effects output by the model have partially indirectly reflected the influence of historical health technology advancements. For instance, the promotion of vaccination and the application of targeted drugs in the past 20 years have led to a reduction in mortality rates for related diseases, which is reflected in the changing trend of the period effects. However, for future technological breakthroughs that have not yet occurred, the model cannot capture them in a forward-looking manner. This is a limitation that we need to clearly acknowledge in our research.

## 4 Discussion

Based on the 2021 GBD database, this study systematically analyzed the evolutionary characteristics, driving mechanisms, and current status of health inequalities in the global burden of CRC from 1990 to 2021 using a multi-dimensional analytical framework. It also predicted future trends with the BAPC model. The core findings not only provide evidence-based support for global CRC prevention and control but also reveal the in-depth impact of socio-economic development and demographic transition on the disease burden.

The significant increase in the global number of CRC incidences, deaths, and DALYs is essentially driven by the “synergistic effect” of population aging and population growth, and this driving effect shows distinct heterogeneity across regions with different SDI. Globally, population aging and population growth contribute 47.43% and 44.85% respectively to the increase in the number of CRC incidences, and their contributions to DALYs are even as high as 71.29% and 75.05%. In contrast, the contribution of epidemiological changes is relatively limited (7.71% to the number of incidences). This is highly consistent with the broader context of global demographic transition [15,16] – as life expectancy increases, the base of the high-risk population for CRC continues to expand, and the growth in total population further amplifies the scale of the disease burden [17]. Notably, there are significant differences in the driving mechanisms between high-SDI and low-SDI regions: in high-SDI regions, the increase in the number of CRC incidences is mainly driven by population growth, and epidemiological changes exert a significant negative inhibitory effect on the number of deaths, which benefits from the mature CRC screening systems and improved comprehensive treatment capabilities in these regions [6]. In low-SDI regions, however, the contribution rate of population aging to the increase in the number of incidences is significantly higher than that in high-SDI regions, and epidemiological changes lead to a positive accumulation of risks for DALYs. This indicates that low-SDI regions not only face the dual pressures of “accelerated aging + population expansion” but also struggle to effectively curb the disease burden through prevention and control measures due to insufficient medical resources and low screening coverage [18]. Such regional differentiation suggests that global CRC prevention and control should develop differentiated strategies based on the demographic structure and medical resource status of regions with different SDIs [19].

The coexistence of alleviation and persistence of health inequalities highlights the structural contradictions in the global CRC prevention and control system [20]. Through the combined assessment using the SII and CI, global health inequalities in CRC improved significantly from 1990 to 2021: the SII decreased from 310 to 192, and the CI decreased from 0.22 to 0.13. This achievement is attributed to the advancement of global public health cooperation, such as the promotion of CRC prevention and control technologies led by relevant organizations and project funding from international institutions for low-SDI regions. Nevertheless, the DALY rate in low-SDI regions remains significantly higher than that in high-SDI regions. This persistent gap is not merely a medical issue but a reflection of the imbalance in the social determinants of health – in low-SDI regions, the low health literacy of residents leads to low rates of early medical consultation, the “treatment-focused over prevention” orientation of medical resources marginalizes screening services, and low economic levels restrict access to treatment [6,11]. More notably, the frontier analysis revealed a phenomenon of “mismatch between efficiency and SDI”: although high-SDI countries such as Monaco and Norway have a relatively low overall disease burden, their effective difference values reach 491.44 and 472.15 respectively, indicating problems such as uneven screening coverage and inefficient resource allocation in their medical systems [6]. In contrast, low-SDI countries like Somalia and the Gambia have achieved near-theoretically optimal prevention and control efficiency through low-cost strategies such as the popularization of primary prevention and the coordinated prevention and control of infectious diseases and tumors. This finding breaks the inherent perception that “high SDI equals high efficiency”, providing a feasible “lean prevention and control” model for low-SDI regions and pointing out the direction for optimizing resource allocation in high-SDI regions. Previous studies have shown that universal screening, early intervention, and optimized treatment plans can significantly reduce the burden of colorectal cancer and improve patient survival rates [21]. Several European cohort studies have demonstrated that national screening programs can reduce CRC mortality by more than 30% [22,23].

The prediction results of the BAPC model bring both warnings and opportunities for global CRC prevention and control. The study predicts that by 2040, the global ASMR of CRC will decrease to 11.05 per 100,000 people, while the number of deaths will increase to 1,013,886. This contradictory trend of “decreasing mortality rate but increasing number of deaths” is mainly due to the combined effect of population aging and population growth – even though advances in medical technology reduce the individual risk of death, the continuously expanding base of high-risk populations will still push up the total number of deaths [24,25]. Meanwhile, predictions regarding gender differences show that the age-standardized incidence rate of CRC in men is significantly higher than that in women. This may be related to factors such as the cancer-inhibiting effect of estrogen, higher occupational exposure risks for men, and lower compliance with healthy behaviors [25–31]. These prediction results suggest that future CRC prevention and control should balance the dual goals of “reducing risks” and “controlling population scale”: for high-SDI regions, priority should be given to strengthening the prevention and control of early-onset CRC to address the younger trend of CRC incidence caused by “Westernized” lifestyles; for low-SDI regions, efforts should focus on expanding screening coverage and improving access to basic treatment, while integrating demographic factors into policy-making, such as advancing the screening age in response to the accelerated aging trend.

This study still has certain limitations that need to be addressed in future research. Firstly, the data of the GBD database in low-SDI regions relies on model estimation, and the lack of original data may lead to deviations in the results. For example, the incomplete cancer registration systems in some regions result in underreporting of the number of incidences, which affects the accuracy of the decomposition analysis. Secondly, the study did not conduct an in-depth quantification of the differences in the contributions of specific risk factors across regions with different SDIs, making it difficult to determine the priority of risk management. Finally, the BAPC model assumes that age, period, and cohort effects are independent of each other, without considering the interaction between them, which may have a certain impact on the prediction results. Future studies can optimize data quality by combining regional cancer registration data, incorporate risk factors into the decomposition model, and improve the BAPC model to include interaction effects, thereby further enhancing the accuracy and practicality of the results.

## 5 Conclusion

In conclusion, this study reveals the changing trend of the global burden of CRC disease and its drivers through multi-dimensional analysis, assesses health inequalities among different countries and regions, and provides a scientific basis for future public health policy making. Future studies should further explore early prevention and screening strategies for CRC, especially in areas with low SDI, and should strengthen the allocation of medical resources and the implementation of public health policies to reduce the disease burden of CRC.

## Supporting information

S1 Table Decomposition analysis of inequality in colorectal cancer deaths counts from 1990–2021 by global and SDI regions. (PDF)

S2 Table Decomposition analysis of inequality in colorectal cancer DALYs from 1990–2021 by global and SDI regions. (PDF)

S1 Fig Gender-specific bayesian age-period-cohort (BAPC) projection of colorectal cancer incidence and mortality rates through 2040. (TIF)
