# Supplementary material for: Multi-dimensional analysis of the global burden of colorectal cancer disease from 1990 to 2021 and prediction of future trends: A comprehensive study based on the GBD database
Source: PLoS One. 2025 Dec 10;20(12):e0337216. doi: 10.1371/journal.pone.0337216 (PMC12694799; doi:10.1371/journal.pone.0337216)
Supplement: S1 Table — (PDF) [file pone.0337216.s001.pdf]

S1 Table: Decomposition Analysis of Inequality in CRC Deaths Counts from 1990-2021 by Global and SDI Regions

Location	Overll difference	Aging	Population	Epidemiological Change	Aging Percentage	Population Percentage	Epidemiological Change Percentage
Global	473753.66	276376.29	388798.73	-191421.36	58.34	82.07	-40.41
High SDI	93348.67	60228.75	143965.04	-110845.12	64.52	154.22	-118.74
High-middle SDI	136952.66	-90619.51	153554.48	74017.69	-66.17	112.12	54.05
Middle SDI	169146.59	88490.67	86960.25	-6304.34	52.32	51.41	-3.73
Low-middle SDI	57386.75	22191.68	28271.01	6924.06	38.67	49.26	12.07
Low SDI	16385.96	7845.76	11156.28	-2616.07	47.88	68.08	-15.97
Eastern Europe	14276.45	-13989.1	28314.87	-49.33	-97.99	198.33	-0.35
Central Europe	19723.36	-1720.05	20211.6	1231.81	-8.72	102.48	6.25
Oceania	216.35	121.44	127.97	-33.07	56.13	59.15	-15.28
East Asia	164241.97	95305.31	95790.86	-26854.19	58.03	58.32	-16.35
Central Asia	1314.94	350.06	2746.54	-1781.66	26.62	208.87	-135.49
Southeast Asia	54642.36	21824.29	22921.73	9896.34	39.94	41.95	18.11
High-income North America	11901.46	11603.31	40656.33	-40358.18	97.49	341.61	-339.1
Central Latin America	7143.31	2485.5	6003.57	-1345.77	34.79	84.04	-18.84
Australasia	2498.02	2796.66	3537.96	-3836.6	111.96	141.63	-153.59
High-income Asia Pacific	45054.86	27703.5	26967.4	-9616.04	61.49	59.85	-21.34
Caribbean	4409.54	-13173.95	6723.46	10860.04	-298.76	152.48	246.28
Western Europe	20260.81	8372.61	73753.91	-61865.7	41.32	364.02	-305.35
Southern Latin America	7143.31	2485.5	6003.57	-1345.77	34.79	84.04	-18.84
Andean Latin America	4055.28	2009.65	1650.91	394.71	49.56	40.71	9.73

North Africa and Middle East	23286.11	12643.92	11791.65	-1149.46	54.3	50.64	-4.94
South Asia	41661.47	19787.97	20975.95	897.55	47.5	50.35	2.15
Tropical Latin America	21312.37	9662.43	8154.55	3495.39	45.34	38.26	16.4
Western Sub-Saharan Africa	6150.62	2138.54	3311.87	700.21	34.77	53.85	11.38
Southern Sub-Saharan Africa	3904.36	996.86	1875.8	1031.7	25.53	48.04	26.42
Central Sub-Saharan Africa	2192.8	1035.53	1192.15	-34.88	47.22	54.37	-1.59
Eastern Sub-Saharan Africa	8188.71	4014.18	5602.68	-1428.16	49.02	68.42	-17.44
