# Supplementary material for: Multi-dimensional analysis of the global burden of colorectal cancer disease from 1990 to 2021 and prediction of future trends: A comprehensive study based on the GBD database
Source: PLoS One. 2025 Dec 10;20(12):e0337216. doi: 10.1371/journal.pone.0337216 (PMC12694799; doi:10.1371/journal.pone.0337216)
Supplement: S2 Table — (PDF) [file pone.0337216.s002.pdf]

**S2 Table: Decomposition Analysis of Inequality in CRC DALYs from 1990-2021 by Global and SDI Regions**

| Location | Overll difference | Aging | Population | Epidemiological Change | Aging Percentage | Population Percentage | Epidemiological Change Percentage |
|---|---|---|---|---|---|---|---|
| Global | 10004442.46 | 7132633.68 | 7507985.5 | -4636176.72 | 71.29 | 75.05 | -46.34 |
| High SDI | 1361137.88 | 1276577.45 | 2396318.39 | -2311757.96 | 93.79 | 176.05 | -169.84 |
| High-middle SDI | 2663664.65 | 1500671.11 | 2218067.94 | -1055074.4 | 56.34 | 83.27 | -39.61 |
| Middle SDI | 3977684.13 | 2415797.13 | 1910495.58 | -348608.57 | 60.73 | 48.03 | -8.76 |
| Low-middle SDI | 1542822.13 | 727017.81 | 655461.51 | 160342.81 | 47.12 | 42.48 | 10.39 |
| Low SDI | 448971.77 | 288529.13 | 257162.72 | -96720.08 | 64.26 | 57.28 | -21.54 |
| Eastern Europe | 184237.7 | -270551.25 | 541605.36 | -86816.42 | -146.85 | 293.97 | -47.12 |
| Central Europe | 320470.95 | -40681.77 | 358768.66 | 2384.05 | -12.69 | 111.95 | 0.74 |
| Oceania | 6490.19 | 4356.7 | 3158.94 | -1025.45 | 67.13 | 48.67 | -15.8 |
| East Asia | 3457443.81 | 2283562.86 | 2058811.32 | -884930.38 | 66.05 | 59.55 | -25.59 |
| Central Asia | 29578.28 | 29976.05 | 62036.02 | -62433.79 | 101.34 | 209.74 | -211.08 |
| Southeast Asia | 1430828.54 | 674409.56 | 519142.21 | 237276.77 | 47.13 | 36.28 | 16.58 |
| High-income North America | 294726.72 | 374856.14 | 708531.8 | -788661.22 | 127.19 | 240.4 | -267.59 |
| Central Latin America | 444841.95 | 202461.85 | 127842.46 | 114537.65 | 45.51 | 28.74 | 25.75 |
| Australasia | 33116.69 | 61216.89 | 61126.77 | -89226.98 | 184.85 | 184.58 | -269.43 |
| High-income Asia Pacific | 567497.74 | 387841.24 | 443323.33 | -263666.84 | 68.34 | 78.12 | -46.46 |
| Caribbean | 96071.99 | 40096.06 | 49060.26 | 6915.67 | 41.74 | 51.07 | 7.2 |
| Western Europe | 184237.7 | -270551.25 | 541605.36 | -86816.42 | -146.85 | 293.97 | -47.12 |
| Southern Latin America | 143343.75 | 62146.2 | 106512.91 | -25315.36 | 43.35 | 74.31 | -17.66 |

| | | | | | | | |
|---|---|---|---|---|---|---|---|
| Andean Latin America | 92195.14 | 51323.7 | 32249.42 | 8622.02 | 55.67 | 34.98 | 9.35 |
| North Africa and Middle East | 602863.35 | 403461.27 | 264722.79 | -65320.71 | 66.92 | 43.91 | -10.84 |
| South Asia | 1121417.51 | 630325.58 | 497665.5 | -6573.57 | 56.21 | 44.38 | -0.59 |
| Tropical Latin America | 526600.18 | 246642.9 | 169346.95 | 110610.33 | 46.84 | 32.16 | 21 |
| Western Sub-Saharan Africa | 166311.56 | 81809.74 | 70976.31 | 13525.51 | 49.19 | 42.68 | 8.13 |
| Southern Sub-Saharan Africa | 105147.47 | 38115.22 | 40870.38 | 26161.87 | 36.25 | 38.87 | 24.88 |
| Central Sub-Saharan Africa | 65618.98 | 38486.48 | 28356.85 | -1224.36 | 58.65 | 43.21 | -1.87 |
| Eastern Sub-Saharan Africa | 218756.63 | 148828.55 | 127624.54 | -57696.46 | 68.03 | 58.34 | -26.37 |
